# Enzymes in Food Processing: A Condensed Overview on Strategies for Better Biocatalysts

**DOI:** 10.4061/2010/862537

**Published:** 2010-09-29

**Authors:** Pedro Fernandes

**Affiliations:** Institute for Biotechnology and Bioengineering (IBB), Centre for Biological and Chemical Engineering, Instituto Superior Técnico, Avenue Rovisco Pais, 1049-001 Lisboa, Portugal

## Abstract

Food and feed is possibly the area where processing anchored in biological agents has the deepest roots. Despite this, process improvement or design and implementation of novel approaches has been consistently performed, and more so in recent years, where significant advances in enzyme engineering and biocatalyst design have fastened the pace of such developments. This paper aims to provide an updated and succinct overview on the applications of enzymes in the food sector, and of progresses made, namely, within the scope of tapping for more efficient biocatalysts, through screening, structural modification, and immobilization of enzymes. Targeted improvements aim at enzymes with enhanced thermal and operational stability, improved specific activity, modification of pH-activity profiles, and increased product specificity, among others. This has been mostly achieved through protein engineering and enzyme immobilization, along with improvements in screening. The latter has been considerably improved due to the implementation of high-throughput techniques, and due to developments in protein expression and microbial cell culture. Expanding screening to relatively unexplored environments (marine, temperature extreme environments) has also contributed to the identification and development of more efficient biocatalysts. Technological aspects are considered, but economic aspects are also briefly addressed.

## 1. Introduction

Food processing through the use of biological agents is historically a well-established approach. The earliest applications go back to 6,000 BC or earlier, with the brewing of beer, bread baking, and cheese and wine making, whereas the first purposeful microbial oxidation dates from 2,000 BC, with vinegar production [1–3]. Coming to modern days, in the late XIX, century Christian Hansen reported the use of rennet (a mixture of chymosin and pepsin) for cheese making, and production of bacterial amylases was started at Takamine (latter to become part of Genencor). Pectinases were used for juice clarification in the 1930s, and for a short period during World War II, invertase was also used for the production of invert sugar syrup in a process that pioneered the use of immobilized enzymes in the sugar industry [1]. Still, the large-scale application of enzymes only became really established in the 1960s, when the traditional acid hydrolysis of starch was replaced by an approach based in the use of amylases and amyloglucosidases (glucoamylases), a cocktail that some years latter would include glucose (xylose) isomerase [1, 2, 4, 5]. From then on, the trend for the design and implementation of processes and production of goods anchored in the use of enzymes has steadily increased. Enzymes are currently among the well established products in biotechnology [6], from US $1.3 billion in 2002 to US $4 billion in 2007; it is expected to have reached US $5.1 billion in a rough 2009 year, and is anticipated to reach $7 billion by 2013 [3, 5, 7–9]. In the overall, this pattern corresponds to a rise in global demand slightly exceeding 6% yearly [7, 9]. Part of this market is ascribed to enzymes used in large-scale applications, among them are those used in food and feed applications [10]. These include enzymes used in baking, beverages and brewing, dairy, dietary supplements, as well as fats and oils, and they have typically been dominating one, only bested by the segment assigned to technical enzymes [11, 12]. The latter includes enzymes in the detergent, personal care, leather, textile and pulp, and paper industries [10, 13]. A recent survey on world sales of enzymes ascribes 31% for food enzymes, 6% for feed enzymes and the remaining for technical enzymes [11]. A relatively large number of companies are involved in enzyme manufacture, but major players are located in Europe, USA and Japan. Denmark is dominating, with Novozymes (45%) and Danisco (17%), moreover after the latter taking over Genencor (USA), with DSM (The Netherlands) and BASF (Germany) lagging behind, with 5% and 4% [10, 11, 14]. The pace of development in emerging markets is suggestive that companies from India and China can join this restricted party in a very near future [15–17].

## 2. Relevant Enzymes: Tapping for Improved Biocatalysts

### 2.1. General Aspects and the Screening Approach

Roughly all classes of enzymes have an application within the food and feed area, but hydrolases are possibly the prevalent one. Representative examples of the enzymes and their role in food and feed processing are given in Table 1. The widespread use of enzymes for food and feed processing is easily understandable, given their unsurpassed specificity, ability to operate under mild conditions of pH, temperature and pressure while displaying high activity and turnover numbers, and high biodegradability. Enzymes are furthermore generally considered a natural product [18, 19]. The whole contributes for developing sustainable and environmentally friendly processes, since there is a low amount of by-products, hence reducing the need for complex downstream process operations, and the energy requirements are relatively low. Life-cycle assessment (LCA) has confirmed, that within the range of given practical case studies, including food and feed processing, the implementation of enzyme-based technology has a positive impact on the environment [3]. LCA is a methodology used to compare the environmental impact of alternative production technologies while providing the same user benefits [20]. 

Some of the broad generalizations on the limitations of enzymes for application as biocatalysts in commercial scale, namely, their high cost, low productivity and stability, and narrow range of substrates, have been rebutted [21, 22]. Aiming at improving the performance of biocatalysts for food and feed applications, particular care has been given to increasing thermal stability, enhancing the range of pH with catalytic activity and decreasing metal ions requirements, as well as to overcoming the susceptibility to typical inhibitory molecules. Some examples of strategies taken to improve the performance of relevant enzymes for food and feed are given in Table 2. Along with these different strategies focused on the enzyme molecule (namely, protein engineering, enzyme immobilization), the developments in recombinant DNA technology that occurred in the 1980s also had a huge impact on the application of enzymes in food and feed. By allowing gene cloning in microorganisms compatible with industrial requirements, this methodology enabled cost-feasible production of enzymes that were naturally produced in conditions that prevented large-scale application (namely, enzymes from plant or animal cells, such as transglutaminase or even slow-growing microorganisms). When successfully implemented, the undertaken approaches allow: (a) continuous operations at relatively high temperatures; (b) eased implementation of enzyme cascade, given the reduced need for processing the reaction media (pH adjustments; metal ion removal/addition) throughout the intermediate steps of a multistep biotransformation (namely, starch to high fructose syrup); and (c) the use of raw substrates, preferably as high-concentrated solutions, hence cutting back in costs related to upstream processing and increasing productivity [4, 23, 24]. Methodologies with a high level of parallelization, anchored in computer-monitored microtiter plates equipped with optic fibers and temperature control have also been developed. These provide high-throughput capability for a speedy and detailed characterization of the performance of enzymes [25]. Particular focus was given to the prediction of the long-term stability of enzymes under moderate conditions using short-term runs (up to 3 hours). 

One of the methodologies to obtain improved biocatalyst relies on in-vitro modifications, which will be addressed latter in this paper; another approach relies on screening efforts, which has been consistently undertaken, as summarized recently [26–31]. Some focus is given to extremophiles, particularly thermophiles, since operation at high temperatures (roughly above 45–50°C) minimizes the risk of microbial contamination, a particularly delicate matter under continuous operation. Furthermore, the extension of some reactions in relevant food applications is favored at relatively high temperatures (namely, isomerization of glucose to fructose), although care should be taken to avoid an operational environment that may lead by-product formation (namely, Maillard reactions). Examples of screened enzymes include the isolation of amylases, with some of them being calcium independent [32–38]; amylopullulanases [39]; fructosyltransferases [40]; glucoamylases [41]; glucose (xylose) isomerases [42, 43]; glucosidases [44, 45]; inulinases [46–49]; levansucrases [50]; pullulanases [51, 52]; and xylanases [53, 54]. Other examples of these enzymes, with some of which able to retain stability under temperatures of 90°C or higher, were reviewed by Gomes and Steiner [55]. The majority of enzymes used in food and feed processing is of terrestrial microbial origin, and screening-efforts for isolation of promising enzyme-producing strains have accordingly been performed in such background [3, 5, 56]. From some years now, marine environment has also been tapped as a source for useful enzymes from either microbial or higher organisms origin [57–60]. This latter environment has allowed the isolation of some promising biocatalysts, such as the heat-stable invertase/inulinase from *Thermotoga neapolitana* DSM 4359 or inulinase from *Cryptococcus aureus* [61–63], amylolytic enzymes, glucosidases and proteases from severalgenera[32, 44, 45, 64, 65], esterase from *Vibrio fischeri* [66],and glycosyl hydrolases [67, 68]. Other examples of useful enzymes for food and feed, but isolated from higher organisms [59, 69], are given in Table 3. Some of these enzymes are actually psychrophiles, hence performing best at low temperatures [30].

Operation at low temperatures is also welcome since it also reduces the risk of microbial contamination, enables some processes to be carried out with minimal deterioration of the raw material. These include protein processing, such as cheese maturing and milk coagulation with proteases [59, 80]; milk processing with lactase for lactose-free milk [81–83]; clarification of fruit juices with pectinases to produce clear juice [84]; or production of oligosaccharides [85].

Since extremophiles are often difficult to grow under typical laboratory conditions if not nonculturable at all, different approaches have been developed in order to assess the potential of enzymes from such microorganisms. One approach relies on the generation and screening of target genes from DNA libraries, which can be obtained from mixed microbial population from environmental samples. Recombinant microorganisms can then be obtained using mesophiles as hosts where the genes of interest from extremophiles have been expressed [86]. In order to screen the huge number of DNA-libraries typically generated for the intended property, high-throughput methods have been implemented [87]. These methods are also widely used when protein engineering is carried out. This will be addressed in the following section. 

Several enzymes (namely, *α*-amylases; pullulanases) currently used in food processing, namely, in starch hydrolysis, are actually produced by recombinant microorganisms. Despite some complexity in the implementation of their use in large-scale applications, partly resulting from lack of uniformity in the US and EU legislation, quite a few enzyme preparations have been accepted for industrial use [88, 89].

## 3. Improving Biocatalysts: Beyond Screening

Taking advantage of the knowledge gathered on molecular biology, high-throughput processing, and computer-assisted design of proteins, *in-vitro* improvement of biocatalysts have been consistently implemented [90–93]. Some of the research efforts in this area has focused on the biochemical and molecular mechanisms underlying the stability of enzymes from extremophiles [31, 94–96]. Such knowledge is also particularly useful for protein engineering of known enzymes, aiming at enhancing stability without compromising catalytic activity [97]. Enhancing the stability of enzymes is of paramount importance when implementation of industrial processes is foreseen, since it allows for reducing the amount of enzyme used in the process. Given that thermostability is determined by a series of short- and long-range interactions, it can be improved by several substitutions of amino acids in a single mutant, where the combination of each individual effect is usually roughly additive [98]. The targeted improvements have not been restricted to thermostability, but they have also addressed other features, such as broadening the range of pH where the enzyme is active, or lessening the temperature of operation while retaining high activity [91, 99]. 

Two methodologies can be used for protein engineering [97].

- The first is directed evolution of enzymes, through random mutagenesis and recombination, where the environmental adaptation is reproduced in-vitro in a much hastened timescale, towards the optimization of the intended property. In order to control the pathway of the process, either a screening test for the assessed feature is performed after each round of modification, or selective pressure is applied [100–102]. This methodology, which allows for a high throughput, has been extensively applied, aiming for more efficient biocatalysts [103–106]. Some relevant examples in the area of food and feed processing include the following.

1. The first is the enhancement of the activity of the hyperthermostable glucose (xylose) isomerase from *Thermotoga neapolitana* at relatively low temperature and pH, without decay in thermostability [107]. The enzyme from the parent strain is highly active at 97°C, but it retains only 10% of its activity at 60°C, and requires neutral pH for optimal activity. This pattern is often reported when glucose isomerases from hyperthermophilic strains operate in mesophilic environments. Large-scale glucose isomerization is carried out at 55–60°C and slightly alkaline pH [1, 31]. This set of conditions results from the optimal range of pH (typically 7.0 to 9.0) and temperature (60 to 80°C) for glucose isomerization displayed by most of the glucose isomerases used, combined with process boundary conditions. The latter result from by-product and color formation occurring when the reaction is carried out at alkaline pH and high temperatures [31, 108]. There is therefore interest in selecting an enzyme able to operate efficiently at temperatures close to those currently used but at a lower pH. The mutant glucose isomerase 1F1 obtained by Sriprapundh and coworkers displayed a roughly 5-fold higher activity at 60°C and pH 5.5, when compared with the parent *T. neapolitana *isomerase, and was more thermostable than the wild type isomerase [104, 107]. The activation energy required by the triple 1F1 mutant (V185T/L282P/F186S) was roughly half of the wild-type, hence allowing for high activity at relatively low temperatures [107]. The encouraging results obtained suggest the soundness of the approach to obtain a mutant glucose isomerase competitive with those currently used, while being able to operate in a slightly acidic environment and 60°C.
2. The second is the enhancement of the thermostability of the maltogenic amylase from *Thermus* sp. IM6501 [109], of the amylosucrase from *Neisseria polysaccharea* [110], of the glucoamylase from *Aspergillus niger* [111], of a phytase from *Escherichia coli* [112, 113], and of a xylanase from *Bacillus subtilis* [114]. Amylases and glucoamylases are enzymes used in starch processing, which involves temperatures typically in excess of 60°C; hence, improving thermal stability without decreasing enzyme activity is of relevance. Starch liquefaction is performed at 105°C in the presence of *α*-amylase, upon which the effluent reaction stream has to be cooled to 60°C, so that glucoamylases can be used. In order to avoid, or at least minimize, the cooling step, thermostable glucoamylases are aimed at. Wang and coworkers obtained a multiply-mutated enzyme (N20C, A27C, S30P, T62A, S119P, G137A, T290A, H391Y), which displayed a 5.12 kJ mol^−1^ increase in the free energy of thermal inactivation, when compared to the wild type, thus resulting in the enhanced thermal stability of the mutant. Furthermore specific activities and catalytic efficiencies remained unaltered, when mutant and wild type were compared [111]. Kim and coworkers obtained also a multiply-mutated amylase (R26Q, S169N, I333V, M375T, A398V, Q411L, P453L) which displayed an optimal reaction temperature 15°C higher than that of the wild-type and a half-life of roughly 170 min at 80°C, a temperature at which the wild-type ThMA was fully inactivated in less than 1 minute. However, one of the mutations most accountable for enhanced thermal stability, M375T, close to the active site, also led to a 23% decrease in specific activity, as compared to the wild type [109]. The amylosucrase engineered by Emond and coworkers was a double mutant (R20C/A451T), displaying a 10-fold increase in the half-life at 50°C compared to the wild-type enzyme. Actually, the mutant was claimed to be the only amylosucrase usable at 50°C. At the latter temperature, the mutant enabled the synthesis of amylose chains twice as long as those obtained by the wild-type enzyme at 30°C, for sucrose concentrations of 600 mM. The mutant thus allowed for a process with increased yield in amylose chains (31 g L^−1^), lower risk of contamination, enhanced substrate and product solubility and overall productivity [110]. Phytases are added to animal feeds to improve phosphorus nutrition and to reduce phosphorus excretion, by promoting the hydrolysis of phytate into myoinositol and inorganic phosphate. Thermal stable enzymes are needed, since feed pelleting is carried out at high temperature (60 to 80°C). Phytases produced by thermophiles do not provide a suitable approach, since they have low activity at the physiological temperature of animals [115]. *E. coli* phytases, which are appealing to industrial application, due to the acidic pH optimum, specificity phytate, and resistance to pepsin digestion, were thus engineered in order to improve their thermal stability, without compromising the kinetic parameters. As a result, mutants were obtained, with roughly 20% increased thermostability at 80°C improved overall catalytic efficiency (*k*_cat_, turnover number/*K* *M*, Michaelis constant) within 50 to 150%, as compared to the wild type. No significant changes in the pH activity profile were observed, but for some mutants, containing a K46E substitution, that displayed a decrease in activity at pH 5.0 [112, 113]. Xylanases catalyze the cleavage of *β*1,4 bonds in xylan polymers. Accordingly, these enzymes can be used in dough making, in baking, in brewing and in animal feed compositions. When the latter contain cereals (namely, barley, maize, rye or wheat), or cereal by-products, xylanases improve the break-down of plant cell walls, which favors the ingestion of plant nutrients by the animals and consequently enhances feed consumption and growth rate. Furthermore, the use of xylanases decreases the viscosity of xylan-containing feeds [116, 117]. As referred for phytases, the formulation of commercial feed often involves steps at high temperatures. Xylanases added to the the formulations hence have to withstand these conditions, while they are to display high activity at about 40°C, which is the temperature in the intestine of animals. However, most xylanases are inactive at temperatures exceeding 60°C, hence the need for enhancing thermal stability [114, 117]. Miyazaki and coworkers obtained a triple-mutant xylanase (Q7H, N8F, and S179C) which retained full activity for 2 hours at 60°C, whereas the wild-type enzyme was inactivated within 5 minutes under the same conditions. The mutation also led to a 10°C increase in the optimal temperature for reaction and enhanced activity at higher temperatures, albeit at the cost of decreased activity at lower temperatures, as compared to the wild-type enzyme [114].
3. Third is the enhancement of the activity of the amylosucrase from *Neisseria polysaccharea* [118]. Amylosucrases can be used for the modification or synthesis of amylose-type polymers from sucrose, but their industrial application is somehow thwarted by the low catalytic efficiency on sucrose and by side reactions leading to the formation of sucrose isomers. Van der Veen and co-works engineered mutant enzymes through error-prone PCR that displayed increases in activity up to 5-fold and in overall catalytic efficiency up to 2-fold, when compared to the wild-type enzyme. Furthermore, the mutants were able to produce amylose polymers from 10 mM sucrose on, unlike the wild-type enzyme [118]. Their work provides an illustrative example on the use of random mutagenesis and recombination for the enhancement of the catalytic properties of enzymes with application on food and feed. Another example was provided by Tian and coworkers who engineered a phytase from *Aspergillus niger* 113 through gene shuffling, to obtain mutants with enhanced catalytic properties [119]. Hence, K41E and E121F substitutions allowed for increases in the specific activity of 2.5- and 3.1-fold, and of affinity for sodium phytate, as expressed by decreases in *K* *M* of roughly 35% and 25%, as compared to the wild-type enzyme. Furthermore, the overall catalytic efficiency of the mutants increased 1.4- and 1.6-fold as compared to the wild type.

Other examples can be found elsewhere [120, 121].

(ii) The second methodology underlines that rational pinpoint modifications in one or more amino acids are made, where these changes are predicted to bring along the envisaged improvement in the targeted enzyme function. The alterations promoted are performed based on the growing knowledge on the structure and functions of enzyme. Information on this matter mostly comes from bioinformatics, which provides data on amino-acid propensities and on protein sequences. Adequate processing of the data enable the output of generalized rules predicting the effect of mutations on enzyme properties. Also used are molecular potential functions, which, once implemented, enable the prediction of the effect of mutations in enzyme structure [97]. Computational tools used for enzyme engineering have been recently reviewed [122]. Enzyme engineering through molecular simulations requires structural data from the native enzyme, which can be preferably obtained from crystallography or NMR. Otherwise a model is built based on known enzyme structures with homologous sequences [90]. Computational methods are also welcome in directed evolution, as a tool to better lead the random mutagenesis [97]. Ultimately this approach is put into practice by producing a site-directed mutant, where selected amino acids are replaced with those suggested from the outcome of modeling.
 Some relevant examples of this strategy in the area of food and feed processing are given. These mostly aim to improve thermal stability and/or catalytic efficiency and/or to modify the range of pH/temperature where the enzyme is active—goals that were already referred to when examples of enzyme modifications using random mutagenesis were addressed.

1. The first example underlines the enhancement of the thermostability of the recombinant glucose (xylose) isomerase from *Actinoplanes missouriensis* [123, 124] and of glucose (xylose) isomerase from *Streptomyces diastaticus* [125]; of amylases from *Bacillus *spp. [126, 127]; and of glucoamylase from *Aspergillus awamori* [128]. The mutant isomerase from *A*. *missouriensis* displayed an enhanced thermal stability, alongside with improved stability at different pH, as compared with the original enzyme, with no changes in catalytic properties [123, 124]. The double mutant isomerase (G138P, G247D) displayed a 2.5-fold increase in half-life, and additionally a 45% increase in the specific activity, when compared to the wild type. Such features were ascribed to increased molecular rigidity due to the introduction of a proline in the turn of a random coil [125]. Multiply-mutated amylases obtained by Declerck and coworkers displayed considered enhanced thermal stability. Based on the temperature at which amylase initial activity is reduced by 50% for a 10-minute incubation, this parameter went as high as 106°C, as compared to 83°C for the wild-type strain. Furthermore, the thermal stabilization was not accompanied by a decrease in the catalytic activity [126]. The work by Lin and coworkers on amylase mutants from *Bacillus* sp. strain TS-23 highlighted the relevance of E219 for the thermal stability of the enzyme [127]. The mutated glucoamylases engineered by Liu and Wang allowed to establish the role of several intermolecular interactions in thermal stability of these enzymes. Thermostable enzymes were obtained through the introduction of disulfide bonds in highly flexible region in the polypeptide chain of the enzyme, as well as by the introduction of more hydrophobic residues-stabilized *α*-helices. Data gathered also showed that care had to be taken not to disrupt the hydrogen bond and salt linkage network in the catalytic center as a result of mutagenesis, for this could lead to a decrease in the specific activity and overall catalytic efficiency [128].
2. The second example underlines the enhancement of the pH-activity profile and of the thermostability of phytase from *A. niger*. This was achieved by combining several individual mutations that allowed for mutants that were quite active at pH 3.5. Efficient operation in the stomach of simple-stomached animals where phytate hydrolysis mostly occurs at a pH around 3.5, and the wild type was ineffective, was thus enabled. Furthermore, the hydrolytic activity of the mutants at pH 3.5 exceeded in roughly 1.5-fold that of the parent one at pH 5.5, which was the optimum of the latter. Mutants also retained higher residual activity after incubation within 70 to 100°C, as compared to the wild type. The work demonstrates that cumulative improvements in pH activity and thermostability through mutation are compatible in this phytase; see [129].
3. The third example underlines the modification of the temperature- and pH activity profile of the l-arabinose isomerase from *Bacillus stearothermophilus* US100 [130]. l-Arabinose isomerases catalyze the conversion of l-arabinose to l-ribulose in-vivo, but in-vitro they also isomerize d-galactose into d-tagatose [130]. The latter keto-hexose is being used as a low-calorie bulk sweetener, since its taste and sweetness are roughly equivalent to sucrose, but the caloric value is only 30% of that of sucrose [131, 132]. Although several thermostable l-arabinose isomerases have been isolated and characterized, most of these display an alkaline pH optimum. For industrial application this presents the same drawbacks of by-product and color formation referred to when the random mutation of glucose isomerases was addressed. Hence, again arises the need for enzymes able to isomerize l-arabinose in an acidic environment and at relatively low temperature, 60 to 70°C. Operation within the latter temperature range also rules away the use of divalent ions, which stabilize isomerases at high temperatures [133, 134]. Rhimi and coworkers engineered two individual mutants, harboring each N175H and Q268K mutations. These led to broader optimal temperature range within 50 to 65°C and to enhanced stability in acidic media, respectively, when compared to the wild type. An engineered double mutant, harboring both modifications, displayed optimal activity within a pH range of 6.0 to 7.0 and a temperature range within 50–65°C. Such set of operational conditions matches the targeted goals and again shows that the basis for pH-activity profile and thermostability in l-arabinose isomerase are quite independent and compatible. Cumulative enhancements in both properties in the same enzyme were thus possible [134]. A similar pattern was also observed in the previous example dedicated to a mutant phytase.
4. The fourth example underlines the modification of the product profile of inulosucrase from *Lactobacillus reuteri* [135] and from *B. subtilis* [136]. Inulosucrases are used to synthesize fructooligosaccharides or fructan polymer from sucrose. The transglycosylation catalyzed by the inulosucrase from *L. reuteri* leads to a wide range of fructooligosaccharides alongside with minor amounts of an inulin polymer. In order to minimize the dispersion in the products obtained, mutants R423K and W271N were obtained, which allowed the synthesis of a significant amount of polymer and a lower amount of oligosaccharide, without significantly affecting the catalytic activity, when compared with the wild type. The data gathered showed that the −1 subsite in the inulosucrase from *L. reuteri* has a key role in the determination of the size of the products obtained [135]. Ortiz-Soto and coworkers also showed that the product profile of transfructosylation reactions could be adequately tuned through modification of target residues of an inulosucrase from *B. subtilis*. These authors established the effect of mutations on the reaction specificity (hydrolysis/transfructosylation), molecular weight and acceptor specificity. For example, engineered mutants R360S, Y429N and R433A only synthesized oligosaccharides, whereas the wild type synthesized levan, since the former are more hydrolytic. On the other hand these mutations reduced the affinity for sucrose, and thermal stability, when compared to the wild type [136].
5. The fifth example underlines the enhancement of the product profile of cyclodextrin glycosyltransferases (CGTase) from differentgenera[137, 138]. These enzymes promote the production of cyclodextrins, *α*(1→4) linked oligosaccharides form starch, through an intramolecular transglycosylation reaction. In the process, a starch oligosaccharide is cleaved and cleaved and the resulting reducing-end sugar is transferred to the non-reducing-end sugar of the same chain [137]. The resulting cyclodextrin may consist of six, seven or eight, which are accordingly termed *α*, *β*, or *γ*-cyclodextrin, respectively. Given their ability to form inclusion complexes with small hydrophobic molecules, they are of interest for both industrial and research applications. Wild-type CGTases typically produce a mixture of the three cyclodextrins when incubated with starch. The purification of a given cyclodextrin from the reaction mixture requires several additional steps, including selective complexation with organic solvents, which may prove restrictive for cyclodextrin applications involving human consumption [139, 140]. There is therefore a clear interest in obtaining a mutant CGTase capable of producing a particular type of cyclodextrin in a high rate. Van der Veen and coworkers engineered a double-mutant (Y89D/S146P) of CGTase from *Bacillus circulans* which displayed a 2-fold increase in the production of *α*-cyclodextrin and a marked decrease in *β*-cyclodextrin when compared to the wild type. From the data gathered, the authors suggested that hydrogen bonds (S146) and hydrophobic interactions (Y89), are likely to play a key role in to the size of cyclodextrin products formed, and that changes in sugar-binding subsites −3 and −7 may result in mutant CGTases with altered product specificity [137]. Li and coworkers were also able to obtain CGTase mutants from *Paenibacillus macerans* strain JFB05-01 with increased specificity for *α*-cyclodextrin, through mutations at subsite −3. In particular, double mutant D372K/Y89R displayed a 1.5-fold increase in the production of *α*-cyclodextrin, and a significant (roughly 45%) decrease in the production of *β*-cyclodextrin when compared to the wild-type enzyme [138].

The two methods are not mutually exclusive and methodologies for engineering of enzymes can assemble both strategies [141].

Upon identification of the most adequate enzyme, this can be formulated adequately for better process integration. One of the most widely considered approaches for such formulation is enzyme immobilization.

## 4. Immobilization

There are several issues that can be lined up to sustain enzyme immobilization. It allows for high-enzyme load with high activity within the bioreactor, hence leading to high-volumetric productivities; it enables the control of the extension of the reaction; downstream process is simplified, since biocatalyst is easily recovered and reused; the product stream is clear from biocatalyst; continuous operation (or batch operation on a drain-and-fill basis) and process automation is possible; and substrate inhibition can be minimized. Along with this, immobilization prevents denaturation by autolysis or organic solvents, and can bring along thermal, operational and storage stabilization, provided that immobilization is adequately designed [142, 143]. Immobilization has some intrinsic drawbacks, namely, mass transfer limitations, loss of activity during immobilization procedures, particularly due to chemical interaction or steric blocking of the active site; the possibility of enzyme leakage during operation; risk of support deterioration under operational conditions, due to mechanical or chemical stress; and a (still) relative empirical methodology, which may hamper scale up. Economical issues are furthermore to be taken into consideration when commercial processes are envisaged, although immobilization can prove critical for economic viability if costly enzymes are used. Still, the cost of the support, immobilization procedure and processing the biocatalyst once exhausted, up- and downstream processing of the bioconversion systems, and sanitation requirements have to be taken into consideration. In the overall, the enhanced stability allowing for consecutive reuse leads to high specific productivity (mass_product_^−1^  mass_biocatalyst_^−1^), which influences biocatalyst-related production costs [1, 142]. A typical example is the output of immobilized glucose isomerase, allowing for 12,000–15,000 kg of dry-product high-fructose corn syrup (containing 42% fructose) per kilogram of biocatalyst, throughout the operational lifetime of the biocatalyst [144]. Increased thermal stability, allowing for routine reactor operation above 60°C minimizes the risks of microbial growth, hence leading to lower risks of microbial growth and to less demanding sanitation requirements, since cleaning needs of the reactor are less frequent [1, 144]. A rule of thumb suggesting that the enzyme costs should be a few percent of the total production costs has been established [142]. The half-life of the bioreactor is also a critical issue when evaluating the economical feasibility of a bioconversion process, longer half-lives favoring process economics. Examples of commercial bioreactors depict half-lives of several months to years, and the same packing can work throughout some months to years. Among this group, are immobilized enzyme reactors packed with glucose isomerase for the production of high-fructose corn syrup; lactase for lactose hydrolysis, for the production of whey hydrolysates and for the production of tagatose; aminoacylase for the production of amino acids; isomaltulose synthase for the production of isomaltulose; invertase for the production of inverted sugar syrup; lipases for the interesterification of edible oils, ultimately targeted at the production of trans-free fat, of cocoa butter equivalents, and of modified triacylglycerols; and *β*-fructofuranosidase for the production of fructooligosaccharides [144–146]. On the other hand, despite the technical advantages of immobilization, the large-scale liquefaction of starch to dextrins by *α*-amylases is performed by free enzymes, given the low cost of the enzyme [18].

Immobilization can be performed by several methods, namely, entrapment/microencapsulation, binding to a solid carrier, and cross-linking of enzyme aggregates, resulting in carrier-free macromolecules [142]. The latter presents an alternative to carrier-bound enzymes, since these introduce a large portion of noncatalytic material. This can account to about 90% to more than 99% of the total mass of the biocatalysts, resulting in low space-time yields and productivities, and often leads to the loss of more than 50% native activity, which is particularly noticeable at high enzyme loadings [142]. A broad, generalized overview of the advantages and drawbacks of the different immobilization approaches is given in Table 4. A typical example of the patterns suggested by data in Table 4 was observed by Abdel-Naby when evaluating the immobilization of *α*-amylase through different methods [147]. Details on the different methods, as well as some illustrative examples of their applications, are given hereafter.

Entrapment/(micro)encapsulation, where the enzyme is contained within a given structure. This can be: a polymer network of an organic polymer or a sol-gel; a membrane device such as a hollow fiber or a microcapsule; or a (reverse) micelle. Apart from the hollow fiber, the whole process of immobilization is performed *in-situ*. The polymeric network is formed in the presence of the enzyme, leading to supports that are often referred to as beads or capsules. Still, the latter term could preferably be used when the core and the boundary layer(s) are made of different materials, namely, alginate and poly-l-lysine. Although direct contact with an adverse environment is prevented, mass transfer limitations may be relevant, enzyme loading is relatively low, and leakage, particularly of smaller enzymes from hydrogels (namely, alginate, gelatin), may occur. This may be minimized by previously cross-linking the enzyme with multifunctional agent (namely, glutaraldehyde) [148, 149] or by promoting cross-linkage of the matrix after the entrapment [150]. The use of LentiKats, a polyvinyl-alcohol-based support in lens-shaped form, has been used for several applications in carbohydrate processing. Among these are the synthesis of oligosaccharides with dextransucrase [149], maltodextrin hydrolysis with glucoamylase [151], lactose hydrolysis with lactase [152], and production of invert sugar syrup with invertase [153]. In these processes the biocatalyst could be effectively reused or operated in a continuous manner. Methodologies for large scale production of these supports have been implemented [154, 155]. Flavourzyme, (a fungal protease/peptidase complex) entrapped in calcium alginate [156], k-carragenan, gellan, and higher melting-fat fraction of milk fat [157], was effectively used in cheese ripening, in order to speed up the process, while avoiding the problems associated with the use of free enzyme. These include deficient enzyme distribution, reduced yield and poor-quality cheese, partly ascribed to excessive proteolysis and whey contamination. The enzyme complex is released in a controlled manner due to pressure applied during cheese curd [156].

Calcium alginate beads were also used to immobilize glucose isomerase [158] and *α*-amylase for starch hydrolysis to whey [159]. In the latter work, the authors observed that increasing the concentration of CaCl_2_ and of sodium alginate to 4% and 3%, respectively, enzyme leakage was minimized (a common drawback of hydrogels) while allowing for high activity and stability. This effect was also observed in a previous work where alginate-entrapped inulinase was used for sucrose hydrolysis [160]. The stability of an amylase immobilized biocatalyst was further enhanced with the addition of 1% silica gel to the alginate prior to gelation, as reflected by the use of the biocatalyst in 20 cycles of operation, while retaining more than 90% of the initial efficiency [159]. Several enzymes, namely, chymosin, cyprosin, lactase, Neutrase, trypsin, have also been immobilized in liposomes, [161]. In a particularly favored technique immobilization of enzymes in liposomes, known as dehydration-rehydration vesicles (DRVs), small (diameters usually below 50 nm) unilamellar vesicles (SUVs) is prepared in distilled water and mixed with an aqueous solution of the enzyme to be encapsulated. The resulting vesicle suspension is then dehydrated under freeze drying or equivalent method. Upon rehydration, the resulting DRVs are multilamellar and larger (from 200 nm to a little above 1000 nm) than the original SUVs, and can capture solute molecules [161, 162]. Recent work in this particular application has used lactase as enzyme model and has focused on the optimization and characterization of the liposome-based immobilized system [163, 164]. If liposome-based biocatalysts are used in a process under continuous operation, biocatalyst separation has to be integrated (namely, using an ultra-filtration membrane). In a different concept, based in batch mode, liposome-encapsulated lactase was incorporated in milk. After ingestion, the vesicles are disrupted in the stomach by the presence of bile salts, allowing *in-situ* degradation of lactose [165]. Cocktails of enzymes, namely, Flavourzyme, bacterial proteases and Palatase M (a commercial lipase preparation), were immobilized in liposomes and successfully used to speed up cheddar cheese ripening [166]. Encapsulation in lipid vesicles has been proved a mild method, providing high protection against proteolysis. There is however some lack of consensus on the feasibility of its application on large scale, as well as on the effectiveness of the methodology for controlled release of enzymes [156, 157, 161, 163, 167]. Containment within an ultra-filtration (UF) membrane allows the enzyme to perform in a fully fluid environment; hence, with little loss (if any) of catalytic activity. However, the membrane still presents a boundary for overall mass transfer of substrate/products and enzyme molecules are prone to interact with the membrane material. This feature is enhanced along with the hydrophobicity of the membrane, hence immobilization in membrane devices may have some adsorptive nature, a feature that will be addressed in (ii). Besides, regular replacement of the membrane may be required. Enzyme containment by a membrane has been used for the continuous production of galactooligosaccharides from lactose. The reaction, with up to 80% lactose conversion out of a substrate concentration of 250 gL^−1^, was carried out in a perfectly mixed reactor and enzyme was recovered in a 10 kDa nominal molecular weight cutoff. The resulting product presented some similarities to the commercially available Vivinal prebiotic [168]. Within the same methodology, a hollow-fiber module was used to contain lactase, in order to carry out lactose hydrolysis in continuous operation. A conversion rate close to 95% in skim milk was observed for an initial substrate concentration close to 40 gL^−1^ [169].

Binding to a solid carrier, where enzyme-support interaction can be of covalent, ionic, or physical nature. The latter comprehends hydrophobic and van der Waals interactions. These are of weak nature and easily allow for enzyme leakage from the support, namely, after environmental shifts in pH, ionic strength, temperature or even as a result of flow rate or abrasion. On the other hand, desorption can be turned into an advantage if performed under a controlled manner, since it enables the expedite removal of spent enzyme and its replacement with fresh enzyme [170]. A recent paper by Gopinath andSugunanillustrates the increased trend for leakage when adsorption is compared with covalent binding, using *α*-amylase as model enzyme [171]. Curiously, the first reported application of enzyme immobilization was of invertase onto activated charcoal [172]. Recently invertase was immobilized in different types of sawdust, aiming at its application for sucrose hydrolysis. When wood shavings were used as support, the immobilized invertase retained 90% of the original activity after 20 cycles of 15 minutes, each under consecutive batch operation; and it retained 65% of the original activity after 10 hours of continuous operational regime in a column reactor [173]. Anther example is the immobilization of pectinase in egg shell for the preparation of low-methoxyl pectin. The immobilized biocatalyst could be reused for 32 times at 30°C, and it was used in a fluidized-bed reactor, operated at an optimum flow rate of 5 mL h^−1^ and 35°C [174]. Other examples are the surface immobilizations of *α*-amylase on alumina [175] and in zirconia [176]. Covalent binding is the strongest form of enzyme linking to a solid support. It involves chemically reactive sites of the protein such as amino groups, carboxyl groups, and phenol residues of tyrosine; sulfhydryl groups; or the imidazole group of histidine. The binding can be carried out by several methods; among them are amide bond formation, alkylation and arylation, or UGI reaction. However, this often brings along loss of activity during the process of immobilization, due to support binding to critical residues for enzyme activity, and steric hindrance, among others. Examples include the immobilization of *α*-amylase [177] and of levansucrase [178] on glutaraldehyde-treated chitosan beads, through the glutaraldehyde reaction between the free amino groups of chitosan and the enzyme molecule; the immobilization of pectinase onto Amberlite IRA900 Cl through glutaraldehyde cross-linking [179]; glucoamylase onto dried oxidized bagasse [180], onto polyglutaraldehyde-activated gelatin [181], or onto macroporous copolymer of ethylene glycol dimethacrylate and glycidyl methacrylate through the carbohydrate moiety of the enzyme [182]; glucoamylase or invertase immobilized onto montmorillonite K-10 activated with aminopropyltriethoxysilane and glutaraldehyde [183, 184]; and invertase immobilized on nylon-6 microbeads, previously activated with glutaraldehyde and using PEI as spacer [185, 186]; on polyurethane treated with hydrochloric acid, polyethylenimine and glutaraldehyde [187]; on poly(styrene-2-hydroxyethyl methacrylate) microbeads activated with epichlorohydrin [188]; or on poly(hydroxyethyl methacrylate)/glycidyl methacrylate films [189]. Within this methodology for immobilization, highlight should be given to the introduction of commercial supports (namely, Eupergit, Sepabeads) with a high density of epoxide functional groups aimed at multipoint attachment, typically with the *ε*-amino group of lysine, to confer high rigidity to the enzyme molecule, hence enhancing stabilization [190, 191]. This methodology has been used for lactase immobilization in magnetic poly(GMA-MMA), formed from monomers of glycidylmethacrylate and ethylmethacrylate, and cross-linked with ethyleneglycol dimethacrylate [192]; for the immobilization of cyclodextrin glycosyltransferases to glyoxylagarose supports for the production of cyclodextrins [193]; or for the immobilization of dextransucrase on Eupergit C [194]. Ionic binding to a carrier involves interaction of negatively or positively charged groups of the carrier with charged amino-acid residues on the enzyme molecules [195]. Ionic interaction may be favored if enzyme leakage is not an issue, since it allows for support regeneration, unlike immobilization by covalent binding. Ion-exchanger resins are typical supports for ionic binding; among them are derivatives of cross-linked polysaccharides, namely, carboxymethyl- (CM-) cellulose, CM-Sepharose, diethylaminoethyl- (DEAE-) cellulose, DEAE-Sephadex, quaternary aminoethyl anion exchange- (QAE-) cellulose, QAE-dextran, QAE-Sephadex; derivatives of synthetic polymers, namely, Amberlite, Diaion, Dowex, Duolite; and resins coated with ionic polymers, namely, polyethylenimine (PEI) [196]. Recent examples include the immobilization of invertase in Dowex [197], in Duolite [198], in poly(glycidyl methacrylate-co-methyl methacrylate beads grafted with PEI [199], and in epoxy(amino) Sepabeads [200]; lactase immobilization in PEI-grafted Sepabeads [201]; fructosyltransferase in DEAE-cellulose for the production of fructosyl disaccharides [202]; glucose isomerase in DEAE-cellulose [203] or in Indion 48-R [204]; glucoamylase onto SBA-15 silica [205] and in epoxy(amino) Sepabeads [200]. Ionic binding to Sepabeads-like supports has acknowledged multipoint attachment nature. Enzyme molecules can be modified chemically or genetically modified to enhance immobilization efficiency, an approach followed by Kweon and coworkers, who obtained a cyclodextrin glycosyltransferase fused with 10 lysine residues to improve ionic binding to SP-Sepharose [206]. 

 Carrier-free macroparticles, where a bifunctional reagent (namely, glutaraldehyde), is used to cross-link enzyme aggregates (CLEAs) or crystals (CLECs), leading to a biocatalyst displaying highly concentrated enzyme activity, high stability and low production costs [142, 207]. The use of CLEAs is favored given the lower complexity of the process. This approach is recent, as compared with entrapment and binding to a solid carrier, and there are still relatively few examples of its application to enzymes used in the area of food processing. Among those are following.

1. First is the immobilization of Pectinex Ultra SP-L, a commercial enzyme preparation containing pectinase, xylanase, and cellulose activities [208]. The CLEA biocatalyst displayed a slight (30%) in the *V*_max_, maximal reaction rate/*K* *M* ratio, but a significant enhancement in thermal stability (a roughly 10-fold increase in half-life), when the pectinase activity of the immobilized biocatalyst was compared with the free form.
2. Second is the immobilization of lactase for the hydrolysis of lactose, where, under similar operational conditions as for the free enzyme, the CLEA yielded 78% monosaccharides in 12 h as compared to 3.9% of the free form [209].
3. Third, CLEAs of glucoamylase, formed by either glutaraldehyde or diimidates, namely, dimethylmalonimidate, dimethylsuccinimidate, and dimethylglutarimidate, led to biocatalysts with improved thermal stability as compared to the free form (over 2-fold increase in half-lives) [210].
4. Fourth, CLEAs of wild type and two mutant levansucrases were assayed for oligosaccharides/levan and for fructosyl-xyloside synthesis. Although the specific activity of the three free enzymes was 1.25- to 3-fold higher than the corresponding CLEAs, these displayed a 40- to 200-fold higher specific activity than the equivalent Eupergit-C-immobilized enzyme preparations. Furthermore, all CLEA preparations displayed enhanced thermal stability when compared with the corresponding free enzymes [211].
5. Fifth are CLECs of glucose isomerase, aimed at the conversion of glucose into fructose for the production of high fructose corn syrup. When placed in a packed-bed, the resulting enzyme preparation allowed for flow rates that matched or even exceeded those processed by commercially available enzyme preparations (either free, carrier free, or carrier-bound), while achieving the same 45% yield in fructose, under similar operational condition [212].
6. Sixth, CLECs of glucose isomerase packed in a column were also used for the concentration/purification of xylitol from dilute or impure solutions. The approach was based on the high specificity of the enzyme crystals towards xylitol, allowing its separation from other sugars, including the natural substrates, xylose and glucose. Recovery of the adsorbed xylitol was achieved by elution with CaCl_2_ solutions, with Ca^2+^ being acknowledged to inactivate glucose isomerase [213].

Each method for enzyme immobilization has a unique nature. Therefore, despite the potential of immobilization to improve enzyme performance by enhancing activity, stability, or specificity, no specific approach tackles simultaneously these different features. A careful evaluation and characterization of the methodology addressed is thus required, which can be significantly fastened by high-throughput approaches [214]. Again, the feasibility of its application to reactor configuration and mode of operation has also to be considered in the selection process of the most adequate immobilized biocatalyst for a given bioconversion. 

### 4.1. Typical Bioreactors

**Figure 1:**
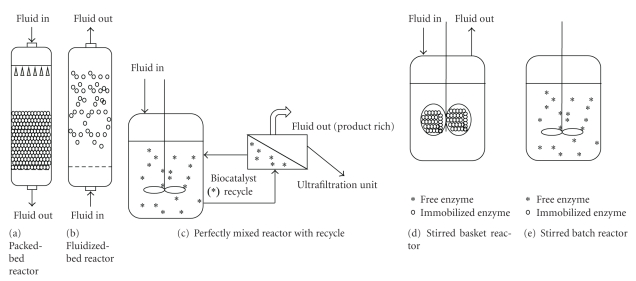
Examples of bioreactor configurations commonly used in bioconversion processed involving free or immobilized enzymes. Reactors (a) to (d) are depicted under continuous mode of operation, whereas reactor (e) is depicted.

The most common form of enzymatic reactors for continuous operation is the packed-bed setup, basically a cylindrical column holding a fixed bed of catalyst particles (Figure 1). These should not have sizes below 0.05 mm, in order to keep the pressure drop within reasonable limits. Commercially available carriers such as Eupergit C have particle sizes of roughly 0.1 mm [215]. Commonly operated in down-flow mode, the range of flow rates used must be such as to provide a compromise between reasonable pressure drop, minimal diffusion layer and high conversion yield. Minimization of external mass-transfer resistances with enhanced flow rates can be considered, leading to the fluidized-bed reactor. This is basically a variation of the packed-bed reactor, but operated in up-flow mode, where the biocatalyst particles are not in close contact which each other; hence, pressure drop is low, and accordingly are pumping costs. The residence time allowed by the flow rates required for fluidization may however result in low conversion yields. This can be overcome by operating a battery of reactor or by operation in recycle mode [216]. Bioconversions with free enzymes are carried out in stirred tanks. When on their own, they are restricted to batch mode, but when coupled to a membrane setup with suitable cutoff, they can be integrated in a continuous process, since the enzymes are rejected by the membrane, which acts as an immobilization device, whereas the product (and unconverted substrate) freely permeates. Shear stress induced by stirring creates a hazardous environment for immobilized biocatalysts, particularly when hydrogels are considered, since they are prone to abrasion. In order to overcome this, a basket reactor was developed, but is seldom used, possibly due to mass transfer resistances associated [18]. 

## 5. Conclusions and Future Perspectives

The integration of enzymes in food and feed processes is a well-established approach, but evidence clearly shows that dedicated research efforts are consistently being made as to make this application of biological agents more effective and/or diversified. These endeavors have been anchoring in innovative approaches for the design of new/improved biocatalysts, more stable (to temperature and pH), less dependent on metal ions and less susceptible to inhibitory agents and to aggressive environmental conditions, while maintaining the targeted activity or evolving novel activities. This is of particular relevance for application in the food and feed sector, for it allows enhanced performance under operational conditions that minimize the risk of microbial contamination. It also favors process integration, by allowing the concerted use of enzymes that naturally have diverse requirements for effective application. Such progresses have been made through the ever-continuing developments in molecular biology, the accumulated evolutionary enzyme engineering expertise, the (bio)computational tools, and the implementation of high-throughput methodologies, with high level of parallelization, enabling the efficient and timely screening/characterization of the biocatalysts. Alongside with these strategies, the immobilization of enzymes has also been a key supporting tool for rendering these proteins fit for industrial application, while simultaneously enabling the improvement of their catalytic features. Again, and despite the developments made in this particular field, there is still the lack of a set of unanimously applicable rules for the selection of carrier and method of enzyme immobilization, which furthermore encompass both technical and economic requirements. The latter can be particularly restrictive in the food and feed sector, since most products are of relatively low added value. Therefore, there is no universal support and method for enzyme immobilization aimed at application in food and feed (let alone the overall range of possible fields of use), and the immobilized biocatalyst fit for a given process and product may be totally unsuitable for another. Given the diversity of enzyme nature and applications this pattern is unlikely to be reversed. Hence, it can be foreseen that efforts will be towards the development of immobilized biocatalyst with suitable chemical, physical, and geometric characteristics, which can be produced under mild condition, that can be used in different reactor configurations and that comply with the economic requirements for large-scale application. All these strategies either isolated or preferably suitably integrated have been put into practice in food and feed, to improve existing processes or to implement new ones, with the latter often combined with the output of new goods, resulting from novel enzymatic activities. Given the recent developments in this field, this trend is foreseen to be further implemented.

## Figures and Tables

**Table 1:** An overview of enzymes used in food and feed processing (adapted from [10, 12, 13, 68]).

Class	Enzyme	Role
Oxidoreductases	Glucose oxidase	Dough strengthening
	Laccases	Clarification of juices, flavor enhancer (beer)
	Lipoxygenase	Dough strengthening, bread whitening

Transferases	Cyclodextrin	Cyclodextrin production
	Glycosyltransferase	
	Fructosyltransferase	Synthesis of fructose oligomers
	Transglutaminase	Modification of viscoelastic properties, dough processing, meat processing

Hydrolases	Amylases	Starch liquefaction and sachcarification
		Increasing shelf life and improving quality by retaining moist, elastic and soft nature
		Bread softness and volume, flour adjustment, ensuring uniform yeast fermentation
		Juice treatment, low calorie beer
	Galactosidase	Viscosity reduction in lupins and grain legumes used in animal feed, enhanced digestibility
	Glucanase	Viscosity reduction in barley and oats used in animal feed, enhanced digestibility
	Glucoamylase	Saccharification
	Invertase	Sucrose hydrolysis, production of invert sugar syrup
	Lactase	Lactose hydrolysis, whey hydrolysis
	Lipase	Cheese flavor, in-situ emulsification for dough conditioning, support for lipid digestion in young animals, synthesis of aromatic molecules
	Proteases (namely, chymosin, papain)	Protein hydrolysis, milk clotting, low-allergenic infant-food formulation, enhanced digestibility and utilization, flavor improvement in milk and cheese, meat tenderizer, prevention of chill haze formation in brewing
	Pectinase	Mash treatment, juice clarification
	Peptidase	Hydrolysis of proteins (namely, soy, gluten) for savoury flavors, cheese ripening
	Phospholipase	In-situ emulsification for dough conditioning
	Phytases	Release of phosphate from phytate, enhanced digestibility
	Pullulanase	Saccharification
	Xylanases	Viscosity reduction, enhanced digestibility, dough conditioning

Lyases	Acetolactate decarboxylase	Beer maturation

Isomerases	Xylose (Glucose) isomerase	Glucose isomerization to fructose

**Table 2:** Some examples of strategies undertaken to improve the performance of enzymes with applications in food and feed.

Enzyme	Role	Targeted improvement	Strategy/comments	Reference
*α*-amylase	Starch liquefaction	Thermostability	Protein engineering through site-directed mutagenesis. Mutant displayed increased half-life from 15 min to about 70 min (100°C).	[70]
	Starch liquefaction	Activity	Directed evolution. After 3 rounds the mutant enzyme from *S. cerevisiae* displayed a 20-fold increase in the specific activity when compared to the wild-type enzyme.	[71]
	Baking	pH-activity profile	Protein engineering through site-directed mutagenesis	[72]

l-arabinose isomerase	Tagatose production	pH-activity profile	Protein engineering through directed evolution	[73]

Glucoamylase	Starch saccharification	Substrate specificity, thermostability and pH optimum	Protein engineering through site-directed mutagenesis	[74]

Lactase	Lactose hydrolysis	Thermostability	Immobilization	[75]

Pullulanase	Starch debranching	Activity	Protein engineering through directed evolution	[76]

Phytase	Animal feed	pH-activity profile	Protein engineering through site-directed mutagenesis	[77]

Xylose (glucose) isomerase	Isomerization/epimerization of hexoses, pentoses and tetroses	pH-activity profile	Protein engineering through directed evolution. The turnover number on D-glucose in some mutants was increased by 30%–40% when compared to the wild type at pH 7.3. Enhanced activities are maintained between pH 6.0 and 7.5.	[78]
		Substrate specificity	Protein engineering through site-directed mutagenesis. The resulting mutant displayed a 3-fold increase in catalytic efficiency with L-arabinose as substrate.	[79]

**Table 3:** Examples of enzymes isolated from various marine higher organisms with potential of application in food and feed (adapted from [68, 69]).

Class	Enzyme	Source
Transferases	Transglutaminase	Muscles of atka mackerel (*Pleurogrammus azonus*), botan shrimp (*Pandalus nipponensis*), carp (*Cyprinus carpio*), rainbow trout (*Oncorhynchus mykiss*), scallop (*Patinopecten yessoensis*).

Hydrolases	Amylase	Gilt-head (sea) bream (*Sparus aurata*), found in Mediterranean sea and coastal North Atlantic Ocean.
		Turbot (*Scophthalmus maximus*), found mostly in Northeast Atlantic Ocean, Baltic, Black and Mediterranean seas, and Southeast the Pacific Ocean
		Deepwater redfish (Sebastes mentella, found in North Atlantic).
	Chymotrypsin	Atlantic cod (*Gadus morhua*), crayfish, white shrimp.
	Pepsin	Arctic capelin (*Mallotus villosus*), Atlantic cod (*Gadus morhua*).
	Protease	Marine sponges *Spheciospongia vesperia*, found in Caribbean sea and South Atlantic, close to Brazil, and *Geodia cydonium*, found in Northeast Atlantic Ocean and Mediterranean sea.
		Mangrove crab (*Scylla serrata*), found in estuaries and mangroves of Africa, Asia and Australia.
		Sardine Orange roughy (*Hoplostethus atlanticus*)

**Table 4:** A generalized characterization of immobilization methods.

Parameter	Immobilization method				
	Carrier binding			CLEAs, CLECs	Entrapment
	Covalent	Ionic	Adsorption		
Activity	High	High	Low	Intermediate/High	High
Range of application	Low	Intermediate	Intermediate	Low	Intermediate/High
Immobilization efficiency	Low	Intermediate	High	Intermediate	Intermediate
Cost	Low	Low	High	Intermediate	Low
Preparation	Easy	Easy	Difficult	Intermediate	Intermediate/Difficult
Substrate specificity	Cannot be changed	Cannot be changed	Can be changed	Cannot be changed	Can be changed
Regeneration	Possible	Possible	Impossible	Impossible	Impossible
